# Portable bacteria-capturing chip for direct surface-enhanced Raman scattering identification of urinary tract infection pathogens

**DOI:** 10.1098/rsos.180955

**Published:** 2018-09-05

**Authors:** Danting Yang, Haibo Zhou, Nicoleta E. Dina, Christoph Haisch

**Affiliations:** 1 Department of Preventative Medicine, Zhejiang Provincial Key Laboratory of Pathological and Physiological Technology, Medical School of Ningbo University, 818 Fenghua Road, Ningbo, Zhejiang Province 315211, People's Republic of China; 2 Department of Pharmacy and Guangdong Province Key Laboratory of Pharmacodynamic of Traditional Chinese Medicine and New Drug Research, Jinan University, Guangzhou, Guangdong Province 510632, People's Republic of China; 3 Department of Molecular and Biomolecular Physics, National Institute of R&D of Isotopic and Molecular Technologies, 67-103 Donat, 400293 Cluj-Napoca, Romania; 4 Chair for Analytical Chemistry, Institute of Hydrochemistry, Technische Universität München, Marchioninistrasse 17, 81377 Munich, Germany

**Keywords:** urinary tract infections, surface-enhanced Raman scattering, rapid detection, chip

## Abstract

Acute urinary tract infections (UTIs) are one of the most common nosocomial bacterial infections, which affect almost 50% of the population at least once in their lifetime. UTIs may lead to lethal consequences if they are left undiagnosed and not properly treated. Early, rapid and accurate uropathogens detection methods play a pivotal role in clinical process. In this work, a portable bacteria-grasping surface-enhanced Raman scattering (SERS) chip for identification of three species of uropathogens (*Escherichia coli* CFT 073, *Pseudomonas aeruginosa* PAO1 and *Proteus mirabilis* PRM1) directly from culture matrix was reported. The chip was firstly modified with a positively charged NH_3_^+^ group, which enables itself grasp the negatively charged bacterial cells through the electrostatic adsorption principle. After the bacterial cells were captured by the chip, concentrated Ag nanoparticles (NPs) were used to obtain their Raman fingerprint spectra with recognizable characteristic peaks and good reproducibility. With the help of chemometric method such as discriminant analysis (DA), the SERS-based chip allows a rapid, successful identification of three species of UTI bacteria with a minimal bacterial concentration (10^5^ cells ml^−1^) required for clinical diagnostics. In addition, this chip could spot the bacterial SERS fingerprints information directly from LB culture medium and artificial urine without sample pre-treatment. The portable bacteria-grasping SERS-based chip provides a possibility for fast and easy detection of uropathogens, and viability of future development in healthcare applications.

## 1. Introduction

Urinary tract infections (UTI) are one of the most common bacterial infections [1] and concern in global healthcare issues. Most of the infections proceed without any complications. However, in some cases, UTI can escalate to bacteraemia, sepsis, renal failure and even death [2]. The most prevalent uropathogenic Gram-negative bacteria are *Escherichia coli*, *Proteus mirabilis* and *Pseudomonas aeruginosa*. Early detection and rapid, precise identification of the causing uropathogens is of great importance in healthcare, pharmacological and biomedical sectors, for longer testing times could lead to a delayed prescription and lethal effects. Currently, the gold standard of diagnosis of UTI, carried out by cultivation, plating and the confirmation of the presence of specific bacteria in urine culture, is time consuming (typically at least 2 days). Alternative test methods directly from urine may apply molecular techniques such as polymerase chain reaction (PCR) or enzyme-linked immunosorbent assays (ELISA). However, PCR and ELISA also involve tedious procedures to some extent (cell lysis, nucleic-acid extraction, and signal amplification for PCR; incubation of antibody and antigen for ELISA), making them impractical for ‘real-time’ detection in the field. Thus, there has always been a strong drive to develop a rapid, sensitive, low-cost, simple, high-throughput approach for clinical bacteria detection. Several advance methodologies including the promising Raman spectroscopy are currently being developed. In addition, the high cost associated with the high-sensitivity commercial PCR test kits or antibody further highlights the advantage of Raman platform [3]. For example, MagMAX™-96 DNA Multi-Sample Kit with 96 Preps (Invitrogen™) costs about US$876 and 100 µl antibody for *E. coli* from Abcam company will cost about US$672, which is not necessary for Raman system.

Usually, identification of UTI bacteria was based on Raman spectroscopy with dielectrophoretic [4–6], or with statistical methodology such as support vector machines [7–9]. However, the fingerprint information of bacteria could not be effectively obtained and thus identifying the variations between spectra of individual cells usually needs more complex classification methods. Surface-enhanced Raman scattering (SERS) distinguishes itself from the above applied techniques by its high speed of analysis, ease of use at low cost, high sensitivity and unique molecular specificity [10,11]. In particularly, SERS is much more sensitive than Raman and has shown its great potential for rapid identification and detection of bacteria [12–16], since the first SERS spectra of bacteria were recorded [17]. However, reports of SERS-based detection of UTI pathogens from complex medium are scarce up to now. Pitris and Ziegler group reported a few proceedings of SERS for classifying bacterial samples as positive or negative for UTI and their antibiotic sensitivities [18,19]. As reported by Avci *et al.* and Mircescu *et al.*, UTI causative bacterial species at their different growth phases could be discriminated [20,21]. The SERS detection of bacteria from complex samples usually needed an enrichment such as filtration and centrifugation or using the help of magnetic nanoparticles (NPs) [22], which takes most time of analytical procedure.

The goal of our study is to construct an easily prepared, portable chip with positive charges to attract negatively charged bacterial cells directly from growing medium without any centrifugation step for rapid SERS detection of UTI bacteria. The feasibility of such chip capable of performing a UTI diagnosis, i.e. (i) identifying the SERS fingerprints of different bacterial species, (ii) classifying the three kinds of UTI bacteria, and (iii) detecting the bacterial cells directly from complex medium, was investigated.

## 2. Material and methods

### 2.1. Chemicals and materials

37% hydrochloric acid (HCl), sodium hydroxide (NaOH), hydroxylamine hydrochloride (NH_2_OH·HCl), 3-glycidyloxypropyltrimethoxysilane (GOPTS) and silver nitrate (AgNO_3_) were purchased from Sigma-Aldrich (Taufkirchen, Germany) and used without further purification. Diamino-PEG 2000 (2000 g mol^−1^) was a gift from Huntsman Holland. LB broth medium, glass slides (26 × 6 × 1 mm), and adhesive tape were purchased from Carl Roth (Karlsruhe, Germany). Ultrapure water (18.2 MΩ cm^−1^) produced using a Millipore water purification system was used as solvent. *E. coli* DSM 1116 shock frozen strains were purchased from DSM nutritional products (Grenzach, Germany). *E. coli* CFT 073, *Ps. aeruginosa* PAO1 and *Pr. mirabilis* PRM1, in LB medium and artificial urine were obtained from the strain collection of the Max-von-Pettenkofer-Institute.

### 2.2. Sample preparation

#### 2.2.1. Concentrated Ag nanoparticles preparation

The stable and highly SERS-active Ag NPs were prepared by reducing a 0.1 mmol silver nitrate solution with 0.17 mmol hydroxylamine hydrochloride by using a modified procedure of Leopold & Lendl [23]. The size and morphology of silver nanoparticles was characterized using a UV–vis spectrometer and transmission electron microscope (TEM). The obtained Ag NPs colloid was then concentrated four times by centrifugation (12 000 r.p.m., 20 min, 4°C). The resulting Ag NPs were shown by Mircescu *et al*. [20] to have higher SERS enhancement for bacteria than the original ones, and were designated as cAg NPs and used in our study.

#### 2.2.2. Bacteria preparation

Three uropathogen species, *E. coli* CFT 073 strain, *Ps. aeruginosa* PAO1 strain and *Pr. mirabilis* PRM1 strain, were obtained from the strain collection of the Max-von-Pettenkofer-Institute. Pure bacterial cultures were grown in 5 ml LB broth culture medium or artificial urine at 37°C under aeration and agitation for 6 h to achieve about 1 × 10^5^ cells ml^−1^ for the following two studies. (i) For the assignment and classification study, in order to avoid the matrix effect on SERS spectra, we firstly harvested the bacterial cells via centrifugation (4500 r.p.m., 15 min, 4°C) and washed by distilled water twice for use. (ii) For the study of bacteria detection directly from LB medium and artificial urine, we directly pipetted the bacterial cells from the culture medium onto the chip without any pre-treatment such as centrifugation and wash step as the above. The stock cell concentration was determined by flow-cytometry using SYTO9. For each bacterial species studied, five independent samples were prepared. For each sample, at least 10 spectra were recorded.

#### 2.2.3. Glass slides treatment

**Figure 1.**
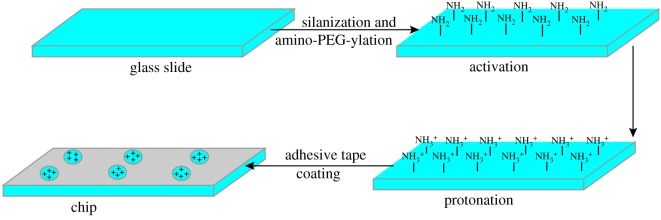
Preparation schematic of portable chip for bacteria-capture and detection.

The portable bacteria-grasping chip preparation consisted of three separate steps, the chip activation, the chip protonation and adhesive tape coating. The preparation schematic is shown in figure 1. Glass slides are chemically and physically stable and exhibit negligible background signals for optical measurements. Firstly, the glass slide was cleaned and activated with active amino sites on the glass surface through silanization and amino-PEG-ylation following the procedure of Wolter *et al*. [24]. Secondly, the slides were immersed in 7.5% HCl aqueous solution for 1 min to form NH_3_^+^ group and then dried under nitrogen flow for further use. Lastly, an adhesive tape (26 × 6 × 1 mm) with six even holes (*r* = 2.3 mm) was placed on the modified glass slide providing the same reaction environment for each hole. The modified glass slides were freshly prepared for further use.

### 2.3. Surface-enhanced Raman scattering measurements

A droplet of 10 µl bacteria sample was pipetted into each hole of the chip to grasp on the chip. Thirty minutes later, 10 µl concentrated Ag NPs (cAg NPs) was added and allowed to interact for another 30 min. Then 10 µl distilled water was added once and removed after 1 min by the pipette to detach the redundant cAg before the SERS measurement. The humidity in the environment is 60%, thus the bacterial cells were not dry when we operated the Raman experiment. The SERS spectra then were recorded with a Raman microscope (LabRAM HR, HORIBA Scientific, Japan) using the 633 nm laser line (He–Ne, 0.14 mW effective power at the sample) and a 50× objective. The holographic grating with 600 lines mm^−1^ yields a spectral resolution of 2 cm^−1^. The exposure time and the accumulation number were set to 1 s and 10, respectively, and the confocal slit width was 100 µm with a spectral region from 50 to 2000 cm^−1^. The wavelength calibration was performed by using a silicon wafer.

### 2.4. Data analysis

#### 2.4.1. Spectral data pre-processing

All the Raman spectra were recorded and processed with LabSpec 5.0 from HORIBA Jobin Yvon and were exported to Origin 2017 to plot. In order to calculate Raman peak intensity, a third order polynomial equation was auto-applied for the intensities of each spectrum and then subtracted from the total spectrum.

#### 2.4.2. Chemometric methods

All the data analysis was carried out using the commercial chemometric software (TQ analyst v. 8.0 from Thermo Fisher). Discriminant analysis (DA) is a supervised pattern recognition algorithm and produces a linear decision boundary between different classes. It has been proven to be effective in many applications and therefore is used in the present study to classify bacterial species.

For discriminant analysis, the Mahalanobis distance of a sample from the centre of gravity of the considered groups was calculated to classify samples as shown in equation (2.1) [25].

2.1 $$\text{MD}={\left(X-X_{\mathrm{avg}}\right)}^{T}V^{-1}(X-X_{\mathrm{avg}}),$$

where MD represents Mahalanobis distance, *X* and *X*_avg_ presents the score vector of sample (*n* × 1) and the mean score vector of sample set (*n* × 1), *V* is the score covariance matrix (*n* × *n*), (*X* − *X*_avg_) and *T* represents the transpose of (*X* − *X*_avg_).

The Mahalanobis distance is a numerical value and used to classify ‘unknown’ individuals to a given species. It is suggested that Mahalanobis distances between two given groups decide the spectral differences between them. The sample can be classified by computing its distance from each class centre of gravity in Mahalanobis distance. If the distance is close to the centre of gravity of its group, the sample is thought to be ‘correctly classified’; otherwise, the sample is ‘poorly classified’ and will be reassigned to the other group.

## 3. Results and discussion

**Figure 2.**
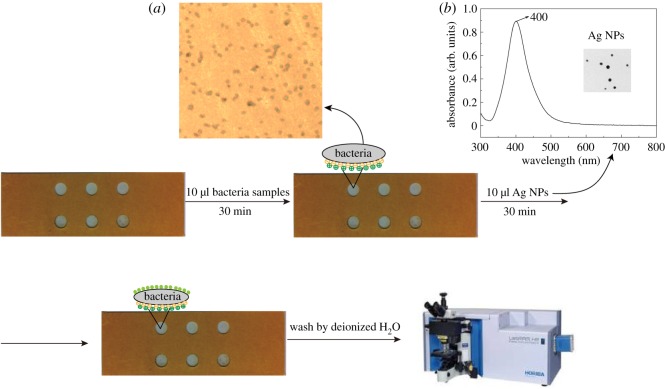
Schematic of designed chip for bacteria capture and detection. (*a*) The optical image of bacteria attached on the chip and (*b*) the UV–vis spectrum and TEM image of Ag nanoparticles (NPs).

The designed chip for bacteria capture and detection is illustrated in figure 2. It is well known that the cell wall of Gram-negative bacteria is negatively charged due to the outer membrane lipopolysaccharides [26]. Hence, bacterial cells can strongly adhere to the surface of glass slides with NH_3_^+^ group by electrostatic interaction after washing twice with distilled water, which was shown in figure 2*a*. The SERS-active substrate Ag NPs are displayed as yellow/greenish suspension and have a narrow absorption peak at 400 nm, indicating monodisperse particles with a diameter of 25 nm (figure 2*b*). After a short incubation with concentrated Ag NPs, Raman signal of bacteria could be enhanced and recorded. In addition, the six identical holes we constructed on the glass slide could capture six bacteria samples simultaneously, which could improve the detection speed for multiple samples.

### 3.1. Surface-enhanced Raman scattering investigation of pure urinary tract infection pathogens

First of all, three representative UTI pathogens such *E. coli* CFT 073 strain, *Ps. aeruginosa* PAO1 strain and *Pr. mirabilis* PRM1 strain were chosen to test the ability of the chip for SERS identification for UTI. In order to avoid the matrix effect on the SERS spectra and obtain clear characteristic peaks, we firstly washed the bacteria cells from the LB medium. One millilitre of three bacteria strains, *E. coli* CFT 073, *Ps. aeruginosa* PAO1 and *Pr. mirabilis* PRM1, were harvested from LB culture medium and washed twice with distilled H_2_O by centrifugation at 4500 r.p.m. and 4°C to remove the LB medium. Then the cell pellet was resuspended in 1 ml of distilled water to remove the LB culture medium. For each species of bacteria, 10 µl bacteria sample of approximately 1 × 10^5^ cells ml^−1^ were pipetted into the hole of chip and incubated for 30 min to ensure the bacteria cells were grasped by the positively NH_3_^+^ group on the surface. Next, 10 µl cAg NPs were added in the hole and allowed to interact with bacteria for 30 min and afterwards washed with 10 µl H_2_O once to remove the free bacteria and Ag NPs. Then Ag NPs would attach closely on the cell wall of the bacteria, and Raman spectra were recorded continuously right after the washing step.

**Figure 3.**
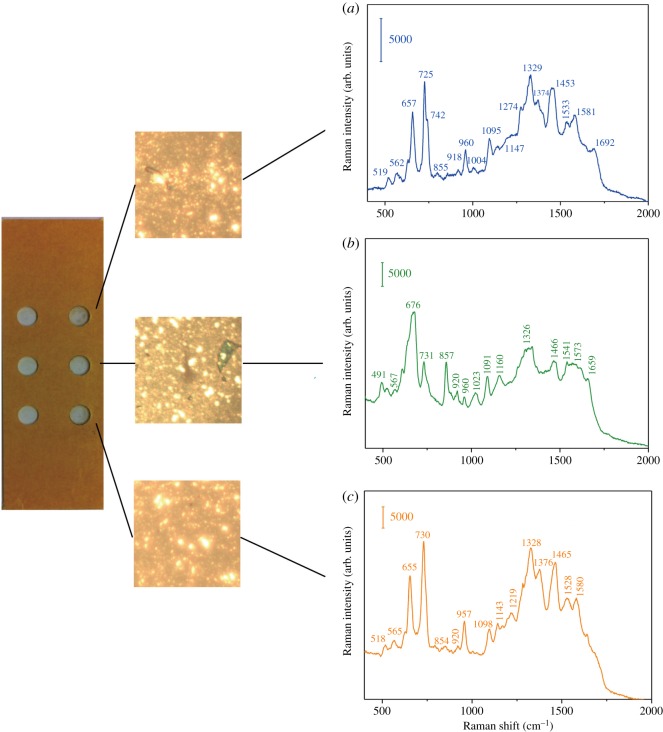
SERS spectra of (*a*) *E. coli* CFT 073, (*b*) *Pr. mirabilis* PRM1 and (*c*) *Ps. aeruginosa* PAO1.

Figure 3 shows the SERS spectra of *E. coli* CFT 073, *Pr. mirabilis* PRM1 and *Ps. aeruginosa* PAO1, respectively. The typical SERS peaks of Gram-negative bacteria at 657, 725, 960, 1095, 1329, 1453, 1581 cm^−1^ and 1692 cm^−1^ with slight variations can be observed, in accordance with the previous report [27,28]. According to the usually invoked molecular origin of SERS spectra of bacteria reported [29–32], the bands are contributed to the major components of the cell wall biopolymer: the δ(COO^−^) and guanine at 657 cm^−1^, adenine or adenine-containing molecules at 725 cm^−1^, υ(CN) at 960 cm^−1^, υ(NH_2_) adenine, polyadenine, adenine at 1329 cm^−1^, δ(CH_2_) saturated lipids 1453 cm^−1^. However, Premasiri *et al.* recently showed the dominant peaks of bacteria were due to the metabolites of purine degradation: adenine, hypoxanthine, xanthine, guanine, uric acid and adenosine monophosphate (AMP) [19,33], which resulted from the starvation response of bacterial cells in pure water washes. The contribution of such purine contribution differs from different bacterial strains. For example, in our case, hypoxanthine, xanthine, guanine, adenine are the largest spectral components of the observed spectra of *E. coli* CFT 073 and *Ps. aeruginosa* PAO1, while guanosine, guanine and xanthine take the most contribution for *Pr. mirabilis* PRM1.

**Figure 4.**
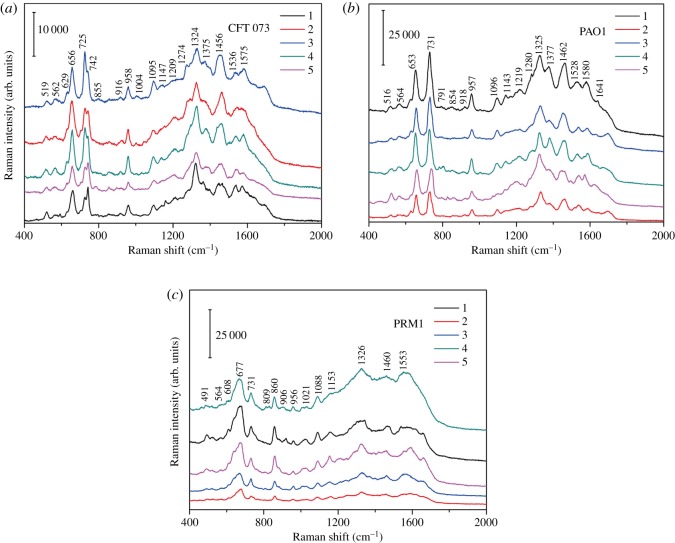
Five SERS spectra of (*a*) *E. coli* CFT 073, (*b*) *Ps. aeruginosa* PAO1 and (*c*) *Pr. mirabilis* PRM1.

The reproducibility of this SERS approach for bacteria characterization is demonstrated by the analysis of five spectra of three bacteria with relative standard deviation (RSD) of (5% for *E. coli*, 6% for *Ps. aeruginosa* and 8% for *Pr. mirabilis*, respectively), indicating this approach highly reproducible and reliable (figure 4).

### 3.2. Discrimination of three urinary tract infection pathogens by surface-enhanced Raman scattering chip

**Figure 5.**
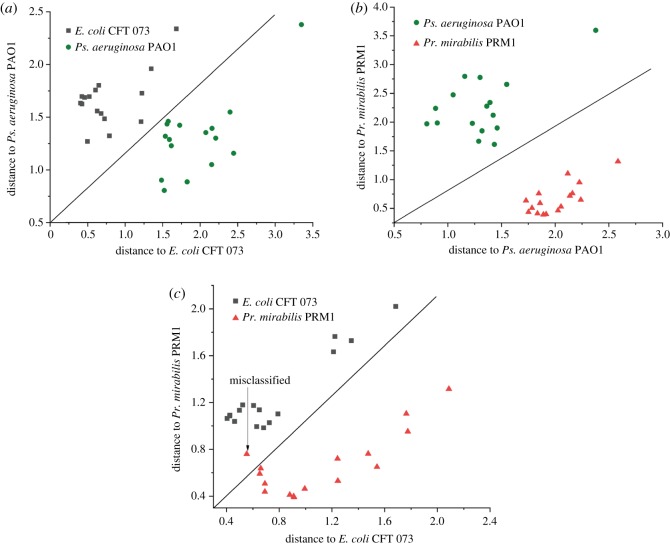
Pairwise Mahalanobis distance of (*a*) ‘*E. coli* CFT 073’, (*b*) ‘*Pr. mirabilis* PRM1’ and (*c*) ‘*Ps. aeruginosa* PAO1’.

To investigate the potential of this chip to discriminate between bacteria by SERS, 48 spectra of samples were chosen (each bacteria species was represented by 16 samples). The SERS spectra of the three uropathogen strains exhibit only minor differences and share some same assignments as shown in table 1. It is difficult to distinguish different species with multiple spectra by naked eye. However, we can discriminate the three bacteria with the help of discriminant analysis (DA) based on a simple algorithm with a whole range from 50 to 2000 cm^−1^ employed. Thirty-six spectra (12 spectra for each bacteria species, respectively) were selected randomly as calibration set. The remaining 12 spectra (four spectra for each bacteria species, respectively) were selected as validation set. The calibration set is used to build classification models, and the validation set is used to validate the performance of calibration models. The Mahalanobis distance plots of every sample to the centre of gravity of two classes (‘*E. coli* CFT 073’ and ‘*Pr. mirabilis* PRM1’ groups, ‘*E. coli* CFT 073’ and ‘*Ps. aeruginosa* PAO1’ groups, as well as ‘*Ps. aeruginosa* PAO1’ and ‘*Pr. mirabilis* PRM1’ groups) by discriminant analysis are shown in figure 5*a–c*. The diagonal line was used to identify the boundary of two classes. From figure 5, it can be seen that the spectra were divided into three clusters according to the species. In the calibration analysis, *Ps. aeruginosa* PAO1 were separated completely from *Pr. mirabilis* PRM1 and *E. coli* CFT 073 groups, as shown in figure 5*a*,*b*. Only one spectrum from *Pr. mirabilis* PRM1 was misclassified as *E. coli* CFT 073, as shown in figure 5*c*. In the validation analysis, all the spectra were correctly classified. The results indicate our SERS-based chip combined with DA method is suitable for the discrimination of three different UTI pathogens.

**Table 1.** The assignments of SERS spectra of *E. coli* CFT 073, *Pr. mirabilis* PRM1 and *Ps. aeruginosa* PAO1 [19,21,33–35].

Raman shift (cm^−1^)			
*E. coli*	*Pr. mirabilis*	*Ps. aeruginosa*	band assignments
519		518	hypoxanthine, guanine
562	567	565	hypoxanthine, xanthine, guanine
657		656	hypoxanthine, xanthine, guanine
	676		guanosine
725	731	730	hypoxanthine, uric acid, adenine guanine, AMP
855	857	854	guanine
960	960	957	hypoxanthine, adenine, guanine, guanosine
	1023		AMP
1095	1091	1098	hypoxanthine
1147		1143	guanosine
	1160		hypoxanthine
		1219	guanine
1274			hypoxanthine, adenine
1329	1326	1328	xanthine, adenine, AMP
1374		1376	hypoxanthine, adenine, AMP
1453	1466	1465	hypoxanthine, adenine, guanine
1533		1528	hypoxanthine
	1541		guanine, guanosine
1581	1573	1580	hypoxanthine, guanosine
	1659	1640	AMP, adenine
1692			hypoxanthine, uric acid

### 3.3. Detection of urinary tract infection pathogen directly from culture medium

**Figure 6.**
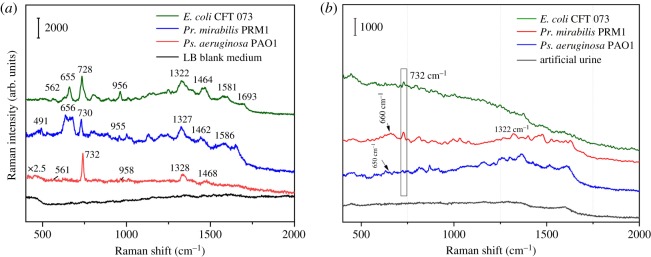
SERS spectra of *E. coli* CFT 073, *Pr. mirabilis* PRM1 and *Ps. aeruginosa* PAO1 in (*a*) LB culture medium and (*b*) artificial urine.

**Figure 7.**
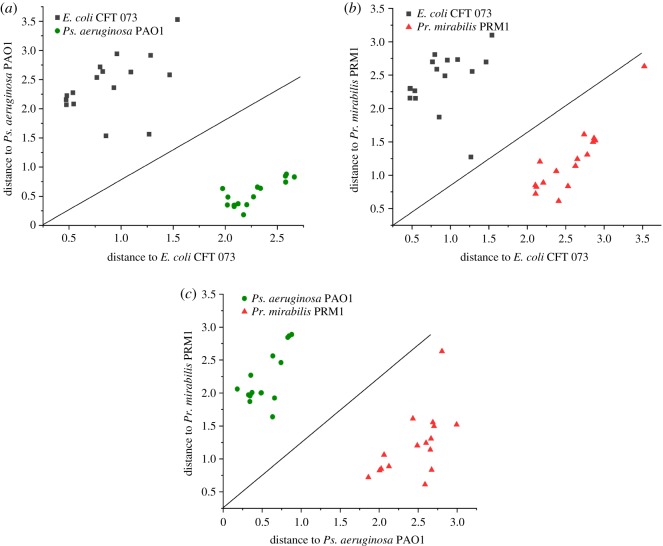
Pairwise Mahalanobis distance of (*a*) ‘*E. coli* CFT 073’, (*b*) ‘*Pr. mirabilis* PRM1’ and (*c*) ‘*Ps. aeruginosa* PAO1’ from LB medium.

The feasibility of this chip we designed for directly detecting uropathogens in complex samples such as LB medium and artificial urine was investigated. Different from the above ‘SERS investigation of pure UTI pathogens’, the bacterial cells in LB medium or artificial urine were directly pipetted onto the chip, without any pre-treatment such as centrifugation and washing with distilled water. Figure 6*a*,*b* shows the SERS spectra of *E. coli* CFT 073, *Pr. mirabilis* PRM1 and *Ps. aeruginosa* PAO1 from LB medium and artificial urine, respectively. The main characteristic peaks of bacterial cells from LB medium could be clearly observed without much difference from the ones centrifuged and washed with distilled water. In addition, the reproducibility of five spectra of three bacteria was calculated with RSD of (6% for *E. coli*, 8% for *Pr. mirabilis* and 9% for *Ps. aeruginosa*, respectively). Besides, in combination with DA method, our portable chip can also successfully discriminate the three kinds of bacteria in LB medium as shown in figure 7 (all the parameters are the same as the above discrimination study). However, fewer bands of bacterial cells from artificial urine can be found. The performance for *Pr. mirabilis* PRM1 was a little better than the other two bacteria with a better appearance at 730 and 1322 cm^−1^. The Raman signal of bacteria from artificial urine was quite different from LB medium, which may be due to different metabolites of purine degradation for different bacterial species [33]. However, the reason needs much more work to find out.

## 4. Conclusion

In this work, we presented a portable bacteria-grasping chip for SERS identification and classification of three kinds of uropathogens. The negatively charged uropathogens could be grasped tightly in the hole on a positively charged glass slide due to the electrostatic adsorption principle. With the help of SERS-active Ag nanoparticles, Raman fingerprints of three uropathogens could be clearly observed and recorded. In addition, the chip allowed itself to classify the three uropathogens within 1.5 h assisted by DA method. Besides, three uropathogens could be spotted directly from LB culture medium without any sample treatment such as filtration and centrifugation, making pre-treatment unnecessary. The six identical holes we constructed on the glass slide could capture six bacteria samples simultaneously, which could improve the detection speed for multiple samples. This portable SERS chip introduces a new possibility for fast and easy detection of uropathogens. However, further research should be carried out to explore a better application in clinical samples. Firstly, the performance of this chip for urine samples should be improved. Secondly, bacterial SERS spectra of more bacterial strains should be collected for constructing a reference data library. Thus, combining chemometric method and reference bacterial SERS database, SERS could be potentially employed in identifying microbes in polymicrobial infections.

## Data accessibility

Our data are deposited at Dryad Digital Repository at: http://dx.doi.org/10.5061/dryad.mr677g8 [36].
